# Favorable neonatal outcome following late-term amniotic membrane rupture with free-floating band: A case report and literature review

**DOI:** 10.1097/MD.0000000000043498

**Published:** 2025-07-18

**Authors:** Lan Wang, Zhengping Wang, Huayun Tan

**Affiliations:** a Obstetrics Medical Center, Weifang People’s Hospital, Shandong Second Medical University, Weifang City, Shandong Province, China; b Ultrasound Department, Weifang People’s Hospital, Shandong Second Medical University, Weifang City, Shandong Province, China.

**Keywords:** amniotic band syndrome (ABS), amniotic rupture sequence (ARS), neonatal outcomes, prenatal diagnosis, ultrasound

## Abstract

**Rationale:**

Amniotic band syndrome (ABS), or amniotic rupture sequence, is a rare congenital condition characterized by fibrous amniotic bands that may entangle fetal parts, leading to constriction, deformities, or even intrauterine demise. However, not all abnormalities of the amniotic membrane result in classical ABS. This case describes a late-term rupture of the amniotic membrane with a free-floating band, but without any evidence of fetal entanglement or malformation. The fetus had a favorable prognosis, suggesting a benign variant of amniotic membrane rupture rather than true ABS.

**Patient concerns:**

A 30-year-old primigravid woman with a history of polyhydramnios, who presented at 39+5 weeks of gestation with concerns of a floating echogenic band suggestive of amniotic membrane rupture and possible umbilical cord entanglement.

**Diagnoses:**

Prenatal ultrasound confirmed persistent polyhydramnios and a ruptured amniotic membrane, with no evidence of constriction around the fetal head or body.

**Interventions:**

Labor was induced at 39 weeks 6 days using intravenous oxytocin, and a vaginal delivery was performed with an episiotomy-assisted approach due to fetal heart rate decelerations.

**Outcomes:**

The infant had Apgar scores of 10 at both 1 and 5 minutes, with normal postnatal growth and development, and the mother recovered well without complications.

**Lessons:**

Timely prenatal diagnosis and careful monitoring are crucial in managing amniotic rupture.

## 1. Introduction

Amniotic band syndrome (ABS) also known as amniotic rupture sequence (ARS) refers to a spectrum of congenital anomalies that arise due to the rupture of the amniotic sac, leading to the formation of fibrous bands that encircle and constrict fetal parts.^[1]^ ABS is typically diagnosed in the first or second trimester via prenatal imaging, and early detection is essential for management.

However, not all amniotic membrane abnormalities result in classical ABS. In rare instances, free-floating amniotic bands may be detected in the third trimester without evidence of fetal constriction or fetal abnormalities. Such findings may represent a variant of amniotic membrane rupture rather than true ABS.

This paper presents a rare case of late-term amniotic membrane rupture with a free-floating band near the umbilical cord, in which no fetal entanglement or malformation occurred. The fetus was delivered healthy at term, and both maternal and neonatal outcomes were favorable. This case highlights the importance of careful differential diagnosis and individualized clinical monitoring when abnormal sonographic findings are encountered in late pregnancy.

## 2. Case presentation

A 30-year-old primigravid woman (gravida 1, para 0) with an unremarkable medical and family history presented at 39+5 weeks of gestation for routine prenatal assessment. At 32 weeks of gestation, she was diagnosed with polyhydramnios, having an elevated amniotic fluid index of 26.7 cm, above the normal range of 5 to 20 cm. However, fetal growth parameters were within normal limits, and no congenital anomalies were observed during prenatal testing, including an NT scan, 4D ultrasound, and noninvasive prenatal deoxyribonucleic acid testing. Despite the polyhydramnios, the fetus showed normal growth, and there were no concerns about congenital defects.

**Figure 1.**
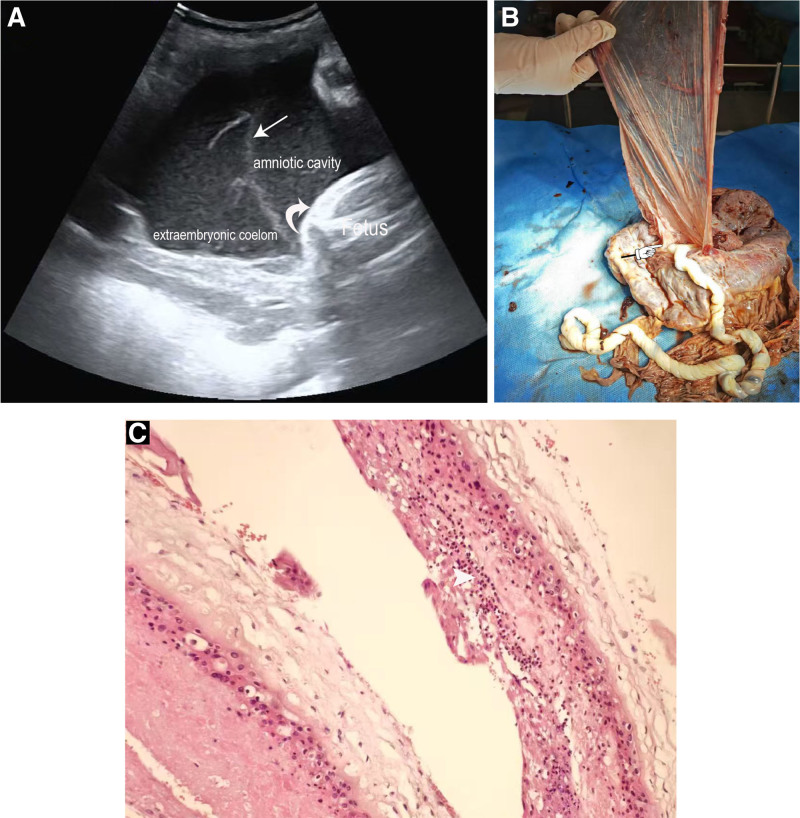
(A) Transabdominal ultrasound image demonstrating a free-floating echogenic amniotic membrane (straight arrow) within the amniotic cavity, adjacent to the extraembryonic coelom, without evidence of constrictive amniotic bands affecting the fetal head or body (curved arrow). (B) Postdelivery photograph illustrating the ruptured amniotic membrane partially encircling the umbilical cord near its insertion site (hand-shaped arrow). (C) Histological section of fetal membranes stained with **Hematoxylin and Eosin (H&E**, 200× magnification), revealing lymphocytic and neutrophilic infiltration consistent with suppurative inflammation (arrowhead).

At 39+5 weeks of gestation, the follow-up ultrasound showed persistent polyhydramnios, with an amniotic fluid index of 27 cm, though still within a moderate range for polyhydramnios (normal range: 8–24 cm). The ultrasound also revealed a floating echogenic band, suggestive of a ruptured amniotic membrane (Fig. 1A). However, there was no evidence of amniotic band constriction around the fetal head or body.

Due to intermittent fetal heart rate decelerations, which raised concerns about possible umbilical cord entanglement and compression, labor was induced at 39+6 weeks using intravenous oxytocin. An episiotomy-assisted vaginal delivery was performed due to the fetal heart rate decelerations. A healthy female infant weighing 2780 g, which was within the expected weight range for the gestational age, was delivered. The infant had an excellent 1-minute Apgar score of 10, indicating no immediate distress or complications, and physical examination showed no abnormal findings.

At the time of delivery, approximately 2500 mL of amniotic fluid was noted, consistent with the prior diagnosis of polyhydramnios. The umbilical cord exhibited 30 spirals, which is within the normal range for healthy cord appearance. Postdelivery examination of the fetal membranes revealed a ruptured amniotic membrane measuring 25 cm × 10 cm, partially encircling the umbilical cord near its insertion site, confirming the ultrasound diagnosis of amniotic membrane rupture (Fig. 1B). Histologic examination of the fetal membranes showed infiltration by lymphocytes and neutrophils, consistent with suppurative inflammation, suggesting a possible infectious or inflammatory process, which could have contributed to the premature rupture of the membranes (Fig. 1C).

The neonate remained clinically stable, demonstrating normal growth and development, with no complications noted in the immediate postnatal period. The mother also recovered well after delivery, and no further complications were noted in her postpartum care. Both the mother and infant had a favorable clinical outcome, with the baby thriving and exhibiting normal growth in the weeks following delivery. Based on the clinical course, imaging, and pathological findings, this case was determined to be a benign variant of amniotic membrane rupture, without evidence of classical ABS.

## 3. Discussion

In this study, we reviewed a collection of ten representative cases of ARS, comparing them to our case. The findings presented in Table 1 illustrate several key similarities and differences between our case and those reported in the literature.^[1–10]^

**Table 1:** Comparison of 10 ARS cases and our case.

Author	Gestational age (wk)	Main ultrasound findings	Fetal outcome
Kalousek DK et al^[1]^	9–20	18 cases with limb and craniofacial anomalies	N/A, emphasized ultrasound importance
Nakashima M et al^[2]^	10, 15, 18	Dividing membrane absent at 15 wk, left arm swelling at 18 wk	Emergency C-section, healthy neonates
Lin HH et al^[3]^	16–37	Five cases with various anomalies (anencephaly, facial clefts, limb abnormalities)	All cases: termination of pregnancy
Sepulveda W et al^[4]^	13	“Turkish turban sign”	Induced delivery, confirmed by autopsy
Hanaoka U et al^[5]^	16	3D ultrasound showed multiple anomalies	Induced delivery, confirmed by autopsy
Schlehe B et al^[6]^	17, 24, 34	Fetal anomalies at 17 wk, fetoscopic intervention at 24 wk	Healthy delivery after intervention
Chen CP^[7]^	12	First-trimester mobile cranial cyst	Termination of pregnancy
Ferreira CR et al^[8]^	36	Multiple anomalies detected by ultrasound	Neonate survived 29 h
Waerlop F et al^[9]^	26	Umbilical cord constriction, fetal distress	Emergency C-section, neonate survived
de Silva MVC et al^[10]^	26	Severe craniofacial anomalies	Infant survived 9 wk
Our case	39+5	Floating amniotic band near umbilical cord, no constriction	Healthy female neonate, Apgar 10, no complications

ARS = amniotic rupture sequence.

This case demonstrates a rare late-gestation amniotic membrane rupture, with free-floating strands contacting the umbilical cord without constriction, causing only transient fetal distress.

Our case demonstrates a rare late-gestation amniotic membrane rupture, with free-floating strands observed near the umbilical cord but without any constriction, deformation, or fetal entanglement-causing only transient fetal heart rate decelerations-and the fetus had a favorable outcome. In contrast, most published ARS and ABS cases involve early amniotic rupture, where fibrous bands form in the first or second trimester, frequently leading to severe fetal outcomes such as limb amputations, craniofacial defects, or intrauterine fetal demise.^[1,3,10]^

Unlike typical umbilical cord amniotic bands, which cause severe compression, fetal hypoxia, and intrauterine demise,^[9]^ our case involved only loose amniotic contact without constriction. Postdelivery pathological examination revealed an umbilical cord with 2 arteries, one vein, perivascular edema, mucinous degeneration, and hemorrhage.

Postdelivery pathological examination revealed an umbilical cord with 2 arteries, 1 vein, perivascular edema, mucinous degeneration, and hemorrhage. These pathological findings are likely the cause of the transient fetal distress. The limited blood flow in the umbilical cord may have impaired oxygen supply to the fetus, resulting in transient distress. Fortunately, timely ultrasound examination and clinical surgical intervention ensured effective management, leading to a positive treatment outcome and favorable prognosis for the newborn.^[5]^

The primary strength of our study lies in the detailed review and comparison of existing case reports, allowing for a comprehensive understanding of the clinical presentation, diagnostic challenges, and outcomes of ARS. Our case adds valuable data on a rare late-term presentation of amniotic membrane rupture, which contrasts with the usual early presentations in previous studies. By including both the clinical and ultrasound findings, we have provided a nuanced view of the potential variability in amniotic membrane rupture presentations.

However, there are limitations to this review. First, the sample size of our case series is limited, and many of the referenced studies include small case numbers. This limits the generalizability of our findings. Additionally, while ultrasound remains a gold standard for diagnosis, the retrospective nature of some of the studies reviewed here may have introduced bias, as diagnoses were made based on postnatal findings or during later stages of pregnancy.

The findings of this review emphasize the importance of early ultrasound detection, particularly for cases of polyhydramnios, which may serve as an early warning sign for amniotic membrane rupture. The ability to diagnose amniotic membrane rupture with precision can significantly influence clinical decisions regarding delivery and postnatal care.

In summary, this case contributes to expanding the diagnostic spectrum of amniotic membrane rupture by illustrating a benign, non-constrictive late-term variant that is clinically and pathologically distinct from classical ABS.

## 4. A patient’s perspective

Timely ultrasound detection, the rich experience of the ultrasound specialist, and surgical intervention were crucial in managing the rare late-gestation amniotic membrane rupture, preventing further complications, and ensuring a positive outcome for me and my child. I am deeply grateful for your timely intervention.

## 5. Conclusion

This study emphasizes the importance of early ultrasound detection and timely surgical intervention in managing late-gestation amniotic membrane rupture. Early diagnosis and appropriate management are crucial for preventing complications and ensuring favorable neonatal outcomes.

## Acknowledgments

The authors express their sincere gratitude to the patient who made this case report possible by providing informed consent and allowing us to share her clinical experience. We would also like to thank the anonymous reviewer(s) for their constructive feedback, which greatly contributed to improving the clarity, accuracy, and overall quality of the manuscript.

## Author contributions

**Conceptualization:** Lan Wang, Zhengping Wang, Huayun Tan.

**Data curation:** Lan Wang.

**Funding acquisition:** Lan Wang.

**Methodology:** Zhengping Wang.

**Resources:** Lan Wang.

**Visualization:** Lan Wang.

**Writing – original draft:** Lan Wang, Zhengping Wang.

**Writing – review & editing:** Lan Wang, Zhengping Wang, Hua yun Tan.
